# Mapping strengths into virtues: the relation of the 24 VIA-strengths to six ubiquitous virtues

**DOI:** 10.3389/fpsyg.2015.00460

**Published:** 2015-04-21

**Authors:** Willibald Ruch, René T. Proyer

**Affiliations:** Personality and Assessment, Department of Psychology, University of ZurichZurich, Switzerland

**Keywords:** character strengths, virtues, VIA-classification, prototypicality, model testing, positive psychology

## Abstract

The Values-in-Action-classification distinguishes six core virtues and 24 strengths. As the assignment of the strengths to the virtues was done on theoretical grounds it still needs empirical verification. As an alternative to factor analytic investigations the present study utilizes expert judgments. In a pilot study the conceptual overlap among five sources of knowledge (strength’s name including synonyms, short definitions, brief descriptions, longer theoretical elaborations, and item content) about a particular strength was examined. The results show that the five sources converged quite well, with the short definitions and the items being slightly different from the other. All strengths exceeded a cut-off value but the convergence was much better for some strengths (e.g., zest) than for others (e.g., perspective). In the main study 70 experts (from psychology, philosophy, theology, etc.) and 41 laypersons rated how prototypical the strengths are for each of the six virtues. The results showed that 10 were very good markers for their virtues, nine were good markers, four were acceptable markers, and only one strength failed to reach the cut-off score for its assigned virtue. However, strengths were often markers for two or even three virtues, and occasionally they marked the other virtue more strongly than the one they were assigned to. The virtue prototypicality ratings were slightly positively correlated with higher coefficients being found for justice and humanity. A factor analysis of the 24 strengths across the ratings yielded the six factors with an only slightly different composition of strengths and double loadings. It is proposed to adjust either the classification (by reassigning strengths and by allowing strengths to be subsumed under more than one virtue) or to change the definition of certain strengths so that they only exemplify one virtue. The results are discussed in the context of factor analytic attempts to verify the structural model.

## Introduction

Both virtues and strengths form essential ingredients of the model of character put forward by Peterson and Seligman (2004). The study of various writings of philosophers and spiritual leaders in China, South Asia, and the West led to the postulate of six ubiquitous core virtues, namely courage, justice, humanity, temperance, wisdom, and transcendence (Dahlsgaard et al., 2005). Virtues are seen as the core characteristics valued by moral philosophers and religious thinkers. Peterson and Seligman (2004) argued that these virtues are universal, perhaps grounded in biology through an evolutionary process that selected for these aspects of excellence as means of solving the important tasks necessary for survival of the species. They chose not to measure the virtues as these are too abstract but put the emphasis on character strengths.

Character strengths were defined as the examples or instances of the virtues. Peterson and Seligman (2004, p. 13) write that character strengths are “[…] the psychological ingredients – processes or mechanisms – that define the virtues. Said another way, they are distinguishable routes to displaying one or another of the virtues. For example, the virtue of wisdom can be achieved through creativity, curiosity etc. […] These strengths are similar in that they all involve the acquisition and use of knowledge, but they are also distinct.” Of lower abstraction is the next level of the good character, namely situational themes. Situational themes are defined as the specific habits that lead people to manifest given character strengths in given situations, be it work-related or in family. The situational conditions that enable or disable strengths have not been studied a lot. Thus, Peterson and Seligman (2004) found it useful to recognize the components of the good character as existing at different levels of abstraction. In this sense the classification scheme is not exclusively horizontal (i.e., distinguishing among virtues or strengths) but also vertical (i.e., specifying different conceptual levels in a hierarchy).

The entries for the classification were found in a separate step. The number of strengths was increased in several steps from 20 to finally 24. In order to qualify as a character strength, a positive trait needed to fulfill several criteria, such as ubiquity (i.e., it is widely recognized across cultures); being fulfilling (i.e., it contributes to individual fulfillment, satisfaction, and happiness broadly construed); being morally valued (i.e., it is valued in its own right and not as a means to an end); not diminishing others (i.e., it elevates others who witness it, producing admiration, not jealousy); having a non-felicitous opposite (i.e., it has obvious antonyms that are “negative,” not also positive); being trait-like (i.e., it is an individual difference with demonstrable generality and stability); measurable (i.e., it has been successfully measured by researchers as an individual difference); its distinctiveness (i.e., it is not conceptually or empirically redundant with other character strengths); the existence of paragons (i.e., it is strikingly embodied in some individuals), and prodigies (i.e., it is precociously shown by some children or youths); the possibility of its selective absence (i.e., it is missing altogether in some individuals, institutions); and larger societies have provided institutions or have developed rituals for fostering the strengths (i.e., it is the deliberate target of societal practices and rituals that try to cultivate it). Peterson and Seligman (2004, p. 18) argue that people typically have between three to seven so-called signature strengths; i.e., “[…] strengths that a person owns, celebrates, and frequently exercises.” Further they list ten possible criteria for a signature strength such as a sense of ownership and authenticity, a feeling of excitement when displaying the strength, or a rapid learning curve for topics associated with the strength. There is broad evidence from placebo-controlled intervention studies that focusing on signature strengths over the course of 1 week has a beneficial impact on happiness and depression (Seligman et al., 2005; for an overview see also Proyer et al., 2015). The final model of strengths and virtues is displayed in **Table 1**.

**Table 1:** The six virtues and 24 character strengths included in the Values in Action classification of strengths and short descriptions defining the strengths and virtues (adapted from Peterson and Seligman, 2004).

(1) *Wisdom and knowledge*: cognitive strengths that entail the acquisition and use of knowledge.

•*Creativity*: thinking of novel and productive ways to do things •*Curiosity*: taking an interest in all of ongoing experience • *Open-mindedness*: thinking things through and examining them from all sides • *Love of learning*: mastering new skills, topics, and bodies of knowledge •*Perspective*: being able to provide wise counsel to others

(2) *Courage*: emotional strengths that involve the exercise of will to accomplish goals in the face of opposition, external or internal.

•*Authenticity*: speaking the truth and presenting oneself in a genuine way •*Bravery*: *not* shrinking from threat, challenge, difficulty, or pain •*Persistence*: finishing what one starts •*Zest*: approaching life with excitement and energy

(3) *Humanity*: interpersonal strengths that involve “tending and befriending” others.

•*Kindness*: doing favors and good deeds for others •*Love*: valuing close relations with others • *Social intelligence*: being aware of the motives and feelings of self and others

(4) *Justice*: civic strengths that underlie healthy community life.

• *Fairness*: treating all people the same according to notions of fairness and justice •*Leadership*: organizing group activities and seeing that they happen •*Teamwork*: working well as member of a group or team

(5) *Temperance*: strengths that protect against excess.

•*Forgiveness*: forgiving those who have done wrong • *Modesty*: letting one’s accomplishments speak for themselves •*Prudence*: being careful about one’s choices; *not* saying or doing things that might later be regretted •*Self-regulation*: regulating what one feels and does

(6) *Transcendence*: strengths that forge connections to the larger universe and provide meaning.

• *Appreciation of beauty and excellence*: noticing and appreciating beauty, excellence, and/or skilled performance in all domains of life • *Gratitude*: being aware of and thankful for the good things that happen •*Hope*: expecting the best and working to achieve it • *Humor*: liking to laugh and tease; bringing smiles to other people • *Spirituality*: having coherent beliefs about the higher purpose and meaning of life

The six core virtues are constituted by three to five character strengths, and the assignment of the strengths to the virtue categories was done on theoretical grounds as opposed to empirically. Later, attempts to examine the model by utilizing factor analysis of the 24 strengths were put forward, but failed to find the proposed six factors. More frequently a solution with five factors has been described—occasionally much to the disappointment of the authors (for a review, see e.g., McGrath, 2014; see also Ruch et al., 2010). In the present manuscript we will first examine the model of the good character closer and then suggest performing an alternative test for its structure.

### Some Testable Assumptions in the Character Model

There are a variety of testable (and partly yet untested) assumptions associated with the VIA-classification. One relates to the condensation of the sources studied by Dahlsgaard et al. (2005) to the “high six.” Would other researchers studying the same writings arrive at the same six core values? Typically, in such a study one would expect an index of convergence among coders. Thus, a valuable but maybe strenuous task would be to go through the literature and cluster the virtues lists or to rate for each of the entries of the virtue lists studied to the degree of prototypicality of each of the virtues. This will help confirming the validity of the selection of virtues in the VIA-model. It should be mentioned that alternative approaches exist to define virtues factors, namely through a psycho-lexical approach (e.g., De Raad and Van Oudenhoven, 2011). Here a longer list of virtue terms from the dictionary is administered to participants for self-report. Virtue factors are then extracted from the intercorrelation of terms administered for self-description. Thus, for example, a virtue factor of “humanity” is dependent on the existence of a sufficient number of other terms that are related to humanity (i.e., related concepts, variants, facets) and of individuals systematically differing in the endorsement of the terms representing these concepts. In other words, humanity, like other classic virtues, would only emerge as a *factor of humanity* if enough *everyday terms* exist that somehow reflect humanity.

A second testable element is the overall relation between strengths/virtues and the “good character.” Peterson and Seligman (2004, p. 13) speculate that all of these virtues must be present at above-threshold values for an individual to be deemed of good character and state that “[a]gain, we regard these strengths as ubiquitously recognized and valued, although a given individual will rarely if ever display all of them. We are comfortable saying that someone is of good character if he or she displays but 1 or 2 strengths within a virtue group.” This assertion contains several elements and challenges. One element to be tested is the perception that the goodness of the character reaches its maximum (and does not progress anymore from there) when all six (rather than merely various combinations of five—or less) virtues are saliently present. This is also based on the assumption that all virtues are needed for a “good character” and that the present list of six is sufficient. In fact, the weight of each of the virtues in the definition of the “good character” could be empirically determined. It will be of interest to see then the distribution of a random sample of adults on such a goodness dimension; i.e., how many have all six virtues above threshold, how many five and so on. A core challenge is to define and validate a criterion for the presence of a strength. What expression of the strengths is needed to speak of “above-threshold values”? Next, the needed critical mass of strengths within a virtue group could be determined empirically as well. Clearly there would be different types of research strategies (e.g., perception studies, predicting criterion behavior) to answer this question. One might also argue that it is not useful to apply the notion of good character to individuals at all but only to the family of strengths and virtues that define it at a conceptual level.

A third possible test examines the relation between character strengths and virtues. As mentioned before, virtually everyone working with the standard instrument for the subjective assessment of character strengths, the *Values-in-Action Inventory of Strengths* (VIA-IS; Peterson et al., 2005), thus far was performing a factor analysis (or principal component analysis, or confirmatory factor analysis) to examine whether the respective strength loads on the virtue factor it was “assigned” to. It should be noted though that nowhere in the original work it is stated that the VIA-classification represents a factor model of character where the virtues are derived from the intercorrelation of the strengths. Occasionally, factor analyses of the strengths are performed with the expectation to arrive at the virtues proposed by Peterson and Seligman (2004), and then the loading of a strength on these factors is taken as a criterion that the strength indeed belongs to the virtue. Peterson and Seligman (2004) do not explicitly specify how their model should be adequately tested, but two strategies seem to be compatible with their writings. Several statements seem to speak against the idea that solely the intercorrelations among the strengths should be used to define the virtues; this is when they write that processes or mechanisms defining the virtues, or distinguishable routes to displaying one or another of the virtues, and that “a given individual will rarely, if ever, display all of them” (Peterson and Seligman, 2004, p. 13). Having to display only one or two strengths in a virtue group implies that being high in some of the strengths of a virtue does not necessarily mean that one needs to be high in other strengths as well to have that strength subsumed under the same virtue. This is plausible, as one might argue that there are several different routes to wealth (such as inheriting money/property, working hard, robbing a bank, gambling successfully, marrying rich, etc.) that do not need to be pursued by the same person to the same extent to make them intercorrelate and form a factor. So these statements clearly indicate that no strict factor model of character is implied. However, in the same book, Peterson and Seligman (2004, p. 26) also mention that correlations with other strengths might serve as a criterion; they note: “We measured only the strengths, and if the data suggest—for example—that playfulness belongs elsewhere because of its co-occurrence with other strengths, we will gladly move it.” Here the other strengths (and the co-occurrence with them) are used as a criterion for belonging rather than its conceptual relation with the virtue (i.e., being a process or mechanism). Nevertheless, while occasionally factor analyses of the VIA-scales have been conducted by these researchers, the outcome of the studies were not meant to change the classification—rather they can be seen as an investigation of the factor structure of a questionnaire. There were discussions to collapse strengths based on the factor analyses to avoid redundancy though (Park and Peterson, 2005). These researchers found four to five factors in the instruments for adults and children/adolescents or a two-factor solution when using ipsative scores (Peterson, 2006).

If factor analysis is not the golden path to test the model what is? For this we need to look into the nature of the model applied. First, it needs to be noted that the hierarchical classification of positive characteristics put forward was modeled on the Linnaean classification of species, which also ranges from the concrete and specific (the individual organism) through increasingly abstract and general categories (population, subspecies, species, genus, family, order, class, phylum, kingdom, and domain). Peterson and Seligman (2004) distinguish the three conceptual levels of situational themes (most specific), character strengths (intermediate), and virtues (most global). Thus, applying Linnaean thinking one would need to specify what attributes of the strengths (or situational themes) might be used in their conceptual classification—and not *individual differences* in the strengths (as essential for factor analytic models). Thus, this assumption needs to be tested conceptually; for example, in a first step by asking experts how good a strength is as an example for each of the six virtues; i.e., into what branch of virtue a strength falls.

The last of the testable assumptions sounds most important as it might provide an alternative to the prior testing of the model. Hence, the present manuscript will examine how good an example each of the 24 strengths is for the six virtues. The major prerequisite for such a study is tested in a pilot study that is aimed at making sure that the different domains of information (e.g., names of the strengths, items, theories of the strengths) are consistent. This is important to know to be able to select the appropriate source of information about a strength to be related to the virtues.

## Pilot Study: Convergence of Indicators of the Strengths

The aim of the pilot study is to examine the degree of conceptual overlap among several domains of information about signature strengths. It is important to ascertain for each character strength that there is a sufficiently high coherence between different sources of knowledge about the strengths. The information examined in the present study includes the names of the strengths including synonyms (e.g., Creativity [originality, ingenuity]), the definitions of the strengths (e.g., thinking of novel and productive ways to do things), brief descriptions of the strengths (e.g., as provided in the feedback to the test takers), a longer theoretical elaboration of the strengths, and the actual item contents. A pairwise comparison among all possible pairs of the five elements will tell whether all five domains cover the strengths (a) sufficiently well, (b) are equal or differently well suited, and (c) whether all strengths are sufficiently coherent, and (d) some strengths are more consistent than others.

## Materials and Methods

### Participants

The sample consisted of 20 German-speaking adults (16 female, 4 male) that served as expert raters. Their mean age was 28 years (ranging from 17 to 49 years). They were either students of psychology or currently working on their Ph.D. in psychology. Eight of them were already familiar with the VIA classification.

### Instruments

#### Strength Definition Comparison Task

Participants were provided with a two page sheet for each strength. The first one contained five different sources of information for the strength, and the second the instruction and 10 rating scales. The five domains of information were (a) the German (and English) names of the scales (including the synonyms), e.g., Creativity (originality, ingenuity), (b) the short definition of the respective strength (e.g., “thinking of novel and productive ways to do things”), (c) a short description of the high scorer (the text was taken from the feedback to the participants that complete the VIA-IS and typically contained between 2 and 5 lines), (d) a more elaborate description of the strengths taken from Peterson and Seligman’s (2004) original work (between 7 and 11 lines), and (e) the actual 10 items from the VIA-IS used to measure this strength. They were instructed to study the five sources and then to perform 10 pairwise comparisons of the five domains regarding the degree of overlap of the two sources. This was done on an 11-point scale with anchored steps (0 = not at all similar, 2 = somewhat similar, but strong conceptual differences, 4 = similar but conceptually different, 6 = similar but not completely identical, 8 = very similar, and 10 = completely identical).

### Procedure and Data Analysis

Data were collected through paper-and-pencil administration. Participants were instructed to do these 240 pairwise comparisons on their own, at their own tempo, and they should take a break if needed. Both the order of strengths and the order of the 10 comparisons of strengths were counterbalanced. Participants were provided with a page with the instructions and the material, and on a separate page they did perform the pairwise comparisons. The task lasted about 2½ h. Participants were not remunerated for their efforts. Data were then averaged to get the 240 mean scores for each pairwise comparison and the strengths. They were then further averaged sequentially (across the comparisons, across strengths) to get scores for the 24 strengths, the five domains, and the 10 types of comparisons.

## Results and Discussion

The grand average of all comparisons was 7.1 indicating that overall there was a considerable overlap, which can be described as being midway between “similar” and “very similar.” This is a high level of coherence in the description of the strengths. At the next lower level of aggregation two types of information can be examined, namely how the conceptual overlap among five sources of knowledge is for each strength (see **Table 2**, last column) and how strongly each of the five domains overlaps on average with the others (see **Table 2**, last row). Finally, the pairwise overlap between the five domains (collapsed over the strengths) is shown in **Table 3**.

**Table 2:** Mean similarity of sources of information about the strength as derived from the pairwise comparisons.

	Label	Definition	Feedback	Description	Items	Total
Perspective	6.4	5.7	6.6	6.6	5.7	6.2
Modesty	6.5	6.0	6.6	6.7	6.4	6.4
Leadership	6.8	5.9	6.8	7.0	6.3	6.5
Love of learning	6.9	5.8	7.0	6.9	6.8	6.7
Creativity	6.9	6.5	7.1	6.7	6.5	6.7
Bravery	6.8	6.7	7.0	6.9	6.5	6.8
Honesty	7.1	6.5	6.8	6.9	6.6	6.8
Love	7.2	6.3	7.0	7.2	6.7	6.9
Teamwork	6.4	6.6	7.0	7.3	7.0	6.9
Kindness	6.8	6.7	7.2	7.0	6.8	6.9
Prudence	7.5	6.8	7.2	7.2	6.5	7.0
Persistence	7.2	6.8	7.1	7.2	7.2	7.1
Curiosity	7.4	6.8	7.4	7.4	7.1	7.2
Self-regulation	7.5	7.0	7.5	7.3	6.9	7.2
Fairness	7.3	7.3	7.4	7.2	7.1	7.3
Hope	7.5	7.3	7.1	7.3	7.2	7.3
Spirituality	7.4	7.1	7.3	7.5	7.2	7.3
Forgiveness	7.7	7.3	7.5	7.1	7.1	7.3
Social Intelligence	7.7	7.0	7.4	7.4	7.2	7.3
Gratitude	7.8	7.2	7.3	7.4	7.3	7.4
Open-mindedness	7.7	7.1	7.6	7.5	7.4	7.5
Beauty and excellence	7.6	7.3	7.6	7.7	7.2	7.5
Humor	7.6	7.2	7.6	7.8	7.7	7.6
Zest	7.9	7.9	7.8	7.7	7.7	7.8

Total	7.2	6.8	7.2	7.2	6.9	7.1

**Table 3:** Mean convergence between the five descriptions of the strengths.

	A	B	C	D	E
A (scale labels)	10.0	6.8	7.5	7.6	7.0
B (one-line definitions)		10.0	7.0	6.8	6.5
C (descriptions in feedback)			10.0	7.3	7.0
D (elaborated descriptions)				10.0	7.2
E (item contents)					10.0

The level of convergence regarding the strengths ranged from 6.2 (i.e., more than “similar”) to 7.8 (i.e., almost “very similar”). This demonstrates that the strengths were more or less consistently described as they are. However, if an improvement in convergence of descriptions is sought, **Table 2** also lists the strengths where improvement is possible (e.g., creativity, love of learning, leadership, modesty, and perspective), while for other strengths (i.e., zest, humor, appreciation of beauty and excellence, open-mindedness, gratitude) the convergence was already very good.

Likewise, the five sources of information about the strengths overlap very well with the others (**Table 2**, last row). Nevertheless, the labels (names, plus synonyms; 7.2), the shorter (7.2) and longer (7.2) descriptions of the strengths yielded high average scores in similarity being again midway between “similar” and “very similar.” The unexpected result was that the labels of the scales yielded the same scores as the description of the strengths (as in the feedback) and the even longer descriptions of the strengths. This might be due to the fact that the words and synonyms are properly chosen and do cover the substance of the strength well. The items yielded a somewhat lower average mean (6.9) and also the one-line *definitions* (6.8) of the strength were somewhat lower. While the variation was not large it is surprising that the items do yield the lowest score.

The inspection of the next lower level of aggregation indicates for each of the 24 strengths how the single sources of information overlap with the four remaining ones. **Table 2** (columns 2–6) confirms that the different levels of information about the strengths do converge to a sufficient to very good level. Most importantly, every single of the 240 comparisons (10 among the five resources, for 24 strengths) was above 5.0 and only four were lower than 6.0. The one-line definitions for perspective, love of learning, and leadership were lower than 6.0, as were the set of items for perspective. All the others at least exceeded the threshold (i.e., exceeded the “similar, but not identical” cut-off score). For zest all five indicators yielded high scores, and while for humor the scores were generally very high, the one-line definition was comparatively lower. For appreciation of beauty and excellence, both the one-line definitions and the items were comparatively lower (albeit still at a very high level of convergence).

Are there some sources of description that systematically converge better than others? **Table 3** shows that the one-line definitions and the item contents not only had the lower scores for convergence, they also had the lowest pairwise overlap (6.5), which, however, is still well beyond cut-off value and can be phrased as “more than similar but not conceptually identical.” The highest overlap was between the labels and the longer descriptions (7.6) and it can be described as close to “very similar” (see **Table 3**).

One might consider it worrying that the items (that constitute the measuring of the strengths in the VIA-IS) yielded lower scores. However, **Table 3** shows that the 10 items not only captured well what is covered in the feedback text and in the longer description, but also converged well with the labels and synonyms. Thus, the labels already capture what is in the items. The one-line definitions converged best with the feedback text, and comparatively lower with the other sources of information. Thus, for the main study the brief descriptions (as used in feedback to participants) will be used to represent the strengths, as they are a good compromise between brevity (of material) and the level of saturation of the respective concept.

## Main study: What Strengths are Prototypical for What Virtues?

Now that it is established that the brief description of the strengths contains the relevant information about the strengths, this layer of information may be used as a representative index in a new study to get estimates of how prototypical each of the strengths is for each of the six virtues. In order to ascertain a standardized understanding of what the virtues are, the raters need to be provided with the definitions of the virtues by Peterson and Seligman (2004) and be instructed to base their judgment on these definitions even if they deviate from their own. Then a rating scale needs to be designed with anchored steps that then allow interpreting the scores. A six-point scale will be utilized and the scale will be anchored in a way, that 3.5 is the cut-off point that needs to be reached to argue that the strengths can be seen as the lower bound of prototypicality for the virtue. A score of 4 will be the lower bound for a good marker and 5 is the cut-off for being a very good marker for the virtue. The rating will include all virtues to be able to see whether the prototypicality is indeed highest for the assigned virtue.

As the six virtues were sought to be representative and distinct, there are reasons to assume that judges will be able to use these scales quite independently form each other and there will be low intercorrelations. However, a correlation between judgments of humanity and justice can be expected and there will also be an overlap between the strengths related to humanity and justice. Peterson and Seligman (2004, p. 293) note: “The entries in this virtue class [humanity] resemble those we identify as justice strengths, with the difference being that strengths of humanity and love are brought to bear in one-to-one relationships, whereas those of justice are most relevant in one-to-many relationships. The former strengths are interpersonal, the latter broadly social.” With regards to justice, Peterson and Seligman (2004, p. 357) argue: “We regard strengths of justice as broadly interpersonal, relevant to the optimal interaction between the individual and the group or the community. As the group shrinks in size and becomes more personalized, the strengths of justice begin to converge with the one-on-one strengths of love discussed in the previous section. We maintain the distinction by proposing that strengths of justice are strengths *among,* whereas those of love are strengths *between,* but the difference is perhaps more of degree than kind.”

This study will use experts of different fields and laypersons to directly estimate to which virtues the strengths belong. While the VIA-classification was based on the discussions among experts (in the think tanks preceding the publication), their number was limited and there was also no report on a formal procedure how agreement was established. Also the experts were mostly psychologists, and one can argue that psychologists are not ideally suited for this task. They might understand strengths well, but virtues were not a topic in psychology at that time, and psychologists might hesitate thinking in terms of virtues, and in particular transcendence is a virtue that is unfamiliar (e.g., Allport, 1937). So it would be good to look for other types of experts as well. Philosophers are familiar with virtue catalogs, but they might doubt the validity of the transcendence category and might also not be that familiar with the strengths concept. Experts might also come from the field of theology and religious studies. They will be familiar with transcendence and not hesitate to give higher prototypicality ratings. One needs to consider that transcendence might mean different things in theology and philosophy. Besides religious studies, there are also hybrid studies, such as psychology of religion, religious education. It will be necessary to represent (personality) psychology, philosophy, and theology to get a more balanced view. Experts should be better in their assignments than laypersons although the average rating of laypersons will be valid as well. Thus, the sample should cover also laypersons and examine how they compare to experts.

It is assumed that experts will assign the strengths to the virtues in the same way as Peterson and Seligman (2004) did. As already stated deviations might occur: some strength might go under a different virtue (e.g., humor maybe may be located under humanity or wisdom, rather than transcendence) or be related to more than one virtue (e.g., social intelligence might be related to humanity and wisdom).

## Materials and Methods

### Participants

The sample consisted 70 experts and 41 laypersons (38.7% women). Their mean age was 41.09 years (SD = 15.95; ranging from 19 to 87 years). Regarding the expert sample larger subsets came from philosophy (*n* = 20), psychology (*n* = 19), theology (*n* = 17), and psychology/pedagogy of religion (*n* = 9).

### Instruments

#### The Strength-Virtue Prototypicality Judgment Instrument

The instrument first contains an instruction followed by definitions of the virtues and the strengths. The descriptions of the virtues were derived from Peterson and Seligman (2004) and varied in lengths with a minimum of 30 and a maximum of 49 words. The description of the strengths were the same as in Study 1. The task was to indicate how good an example^1^ the 24 strengths are for each of the six virtues using a six-point scale (1 = not at all, 2 = marginally, 3 = not very good, 4 = quite good, 5 = good, and 6 = very good). There was also an empty slot to add a further virtue and rate the degree of prototypicality.

### Procedure

The study was conducted via the internet using the SurveyMonkey platform. Experts were either personality psychologists or had a strong link to virtue and came from the following fields: personality, philosophy, ethics, theology, psychology of religion, or pedagogy of theology. They were initially spotted at university websites (departments of psychology, theology, philosophy etc.), membership lists of professional societies and approached via email and asked to participate in the study. About 70% of the experts actually agreed to participate. They typically were professors in the respective field or were holding a Ph.D. and specialized in the field. Laypersons had no particular background in personality and/or virtues. Typically these were students or relatives of students. As a double check all participants were asked to indicate their level of expertise in these fields in the survey. They received no compensation for participating in the study but were promised a written summary on the main findings of the study.

### Data Analysis

The main analysis will be rather simple and involve an averaging of the prototypicality scores and presenting them in a table with 6 virtues and 24 strengths. Applying the cut-off scores one can then see whether a strength is not marker (1.0–3.5) a marker (>3.5), a good marker (>4.0) or a very good marker (>5.0) for a virtue. Then we will count how many of the 24 strengths do actually mark the virtue they are assigned for and at what level. We can also see how often a strength is a marker for a virtue it was not original assigned to. One can also count how often a strength marks more than one virtue, and whether the prototypicality score is higher for the assigned virtue than for another. To examine whether expert status (experts, laypersons) matters and to remove a bias due to age and gender, a 6 × 24 ANCOVA with the six virtues and the 24 strengths on the repeated measure factors and expert status (experts, laypersons) as a grouping variable and gender and age as covariates will be computed for the prototypicality ratings. Of the possible effects only the virtue times strength interaction is of prime interest and some of the effects (e.g., covariates, interaction with covariates) are neglected altogether as the aim is only to get an estimate for a prototypicality score that is unbiased by these variables. Furthermore, similar analyses will be computed to test whether type of raters (type of expert, layperson) matters to then apply the cut-off scores and see how well the strengths mark the virtues. It is not attempted to do *post hoc* tests to see whether mean scores are significantly different (i.e., whether a strength is significantly more prototypical for one virtue than for a different virtue; or whether a virtue is more linked to one strength than to the other strength).

Two analyses will be computed to show the structure inherent in the virtues and in the strength. The results of the analyses should be valid across the other two modes (rater, and strengths or virtues). The correlations of the virtue prototypicality ratings were computed across participants and strengths, but not tested for significance. Here it will not actually be of interest to see whether a correlation is “significant” but to demonstrate that the judgments are relatively independent—with the exception of humanity and justice. Finally, the similarity among the strengths (across raters and virtues) will be examined and analyzed in a lower dimensional space. A principal component analysis will be performed on the intercorrelations among the 24 strengths. The number of factors will be examined using the Scree test and a Varimax rotation will be performed. The resulting matrix allows investigating whether the strengths assigned to a virtue actually form a joint factor when the intercorrelations are based on the prototypicality ratings for the virtues; i.e., strengths load on the same factor when they are rated high on the same virtues. Individuals also enter into the mode analyzed but not regarding how much they possess that strength but how they see the strength-virtue connections.

## Results

### Prototypicality Ratings

The correlations of the prototypicality ratings for the six virtues (across participants and strengths) were computed and are presented in **Table 4**.

**Table 4:** Intercorrelation among the prototypicality ratings for the six virtues.

	Courage	Humanity	Justice	Temperance	Transcendence
Wisdom	0.24	0.23	0.32	0.28	0.27
Courage		0.20	0.34	0.13	0.20
Humanity			0.59	0.34	0.33
Justice				0.41	0.23
Temperance					0.19

*N* = 2664.

**Table 4** shows that all intercorrelations were positive. As expected, the correlations are higher for justice and humanity; i.e., when a strength was considered prototypical for humanity it also tended to be prototypical for justice. High prototypicality for justice was also correlated with the temperance judgments. The other correlations were lower than 0.40. Thus, overall the participants discriminated well among all six virtues and they saw justice somewhat more correlated with both humanity and temperance.

### How Prototypical are the Strengths for the Virtues?

A 6 × 24 ANCOVA with the six virtues and the 24 strengths on the repeated measure factors and expert status (experts, laypersons), gender and age as a grouping variable was computed for the prototypicality ratings. The covariates had no main effects (*p* > 0.14), but were involved in some interactions. The main effect of virtues was not significant [*F*(5,535) = 1.119, *p* = 0.349], and the virtue ratings did not interact with any of the three covariates (*p* > 0.51). There was a main effect for strengths [*F*(23,2461) = 3.414, *p* < 0.001, $\eta_{p}^{2}$ = 0.031], and more importantly a strengths-virtue interaction, *F*(115,12305) = 4.493, *p* < 0.001, $\eta_{p}^{2}$ = 0.040. While the strengths interacted with all covariates (*p*s < 0.012; $\eta_{p}^{2}$ = 0.016–0.022), the virtue × strength interaction did not depend on gender or expert status (*p*s > 0.015), but was moderated by age, *F*(115,12305) = 1.493, *p* < 0.001, $\eta_{p}^{2}$ = 0.014. These effects were not further explored, as they were small and seemed difficult to generalize to other studies. However, they were being controlled for in the mean prototypicality ratings presented in **Table 5**.

**Table 5:** Mean prototypicality of each the 24 strengths for the six virtues (controlled for effects of gender, age, expert status) as well as the overall virtue prototypicality.

	Wisdom	Courage	Humanity	Justice	Temperance	Transcendence	Overall
Creativity	**4.27**	3.78	2.46	1.97	1.78	2.89	2.89
Curiosity	**4.82**	3.80	2.78	2.19	1.84	3.12	3.09
Open-mindedness	**5.44**	3.44	3.13	3.98	2.96	2.66	3.58
Love of learning	**5.31**	2.95	2.71	2.21	2.27	2.87	3.06
Perspective	**5.80**	2.75	3.73	3.85	3.41	3.56	3.83
Bravery	2.73	**5.92**	3.09	3.30	2.58	2.66	3.37
Persistence	3.57	**4.05**	2.56	2.94	3.77	2.40	3.27
Honesty	3.60	**4.26**	3.69	4.15	2.94	2.78	3.59
Zest	2.63	**4.32**	2.68	2.24	1.84	2.78	2.72
Love	2.79	3.06	**5.25**	3.13	3.00	3.49	3.48
Kindness	2.70	2.48	**5.40**	3.10	2.65	3.06	3.22
Social intelligence	4.28	2.72	**4.76**	3.96	3.21	2.51	3.57
Teamwork	3.14	2.62	*4.58*	***4.23***	3.45	2.75	3.51
Fairness	3.43	3.05	4.41	**5.86**	3.36	2.79	3.83
Leadership	*3.93*	*4.14*	3.54	***3.92***	2.92	2.24	3.48
Forgiveness	3.67	3.05	*5.47*	*4.02*	***3.71***	3.65	3.93
Modesty	3.28	2.14	3.70	2.91	**4.76**	3.29	3.36
Prudence	4.14	2.05	2.77	2.76	**4.63**	2.29	3.10
Self-regulation	3.33	2.78	2.69	2.78	**5.70**	2.60	3.32
Beauty	3.63	2.06	2.82	1.91	1.91	**4.26**	2.71
Gratitude	3.21	2.14	*4.57*	3.55	3.06	***3.72***	3.34
Hope	3.08	3.92	3.41	2.68	2.35	**4.10**	3.26
Humor	3.30	2.78	*3.96*	2.09	2.10	***2.47***	2.76
Spirituality	2.83	2.27	3.01	2.58	2.78	**5.72**	3.19

Beauty = Appreciation of beauty and excellence. Virtue assignment in Peterson and Seligman (2004) are shown in boldface; italics indicate strengths that are more prototypical for a virtue other than the assigned one.

**Table 5** shows that nine strengths were very good markers (prototypicality > 5.0) and 11 were good markers (prototypicality > 4.0) for the virtue they were assigned to. Three more exceeded the 3.5 threshold (i.e., were quite good markers) and only humor did *not* reach the threshold for being prototypical for the virtue it was assigned to (i.e., >3.5). Humor seemed to be a marker for humanity. Two other strengths had a numerically higher prototypicality for a different virtue than its own, namely teamwork (for humanity in addition to justice) and gratitude (for humanity in addition to transcendence). For two strengths there were two additional virtues at least equally relevant: forgiveness was more prototypical for humanity and justice than it was for temperance, and leadership was more relevant for courage and for wisdom than it was for justice. It should be mentioned that four strengths (that marked the own virtue best) were also good markers (>4.0) for a further virtue: honesty for justice, social intelligence and prudence for wisdom, and fairness for humanity. Said in a different way, of the strengths that were a very good marker for their virtue only one was also a marker for a different virtue. Of the 11 strengths that were good markers for their virtue four also marked a second virtue. Finally, all three strengths that were quite good markers for their own virtue also were good or very good markers for one or two other virtues. Thus, for seven strengths no “one to one”-correspondence to a virtue could be found but they proved to be more complex. The only strength that was wrongly assigned turned out not to be complex with only being prototypical for humanity.

As there also was a powerful main effect, the overall virtuousness rating will be considered, too (see last column in **Table 5**). It is evident from **Table 5** that the strengths with the lower scores had both a lower prototypicality rating for the assigned virtue and also fewer virtues they were prototypical for. These strengths (i.e., high curiosity, love of learning, creativity, humor, zest, appreciation of beauty and excellence) also did not feel very virtuous compared to the ones with a high total score (i.e., forgiveness, perspective, fairness, honesty, open-mindedness, and social intelligence), which all marked justice and tended to mark humanity and wisdom.

### Does the Nature of the Experts Matter?

**FIGURE 1:**
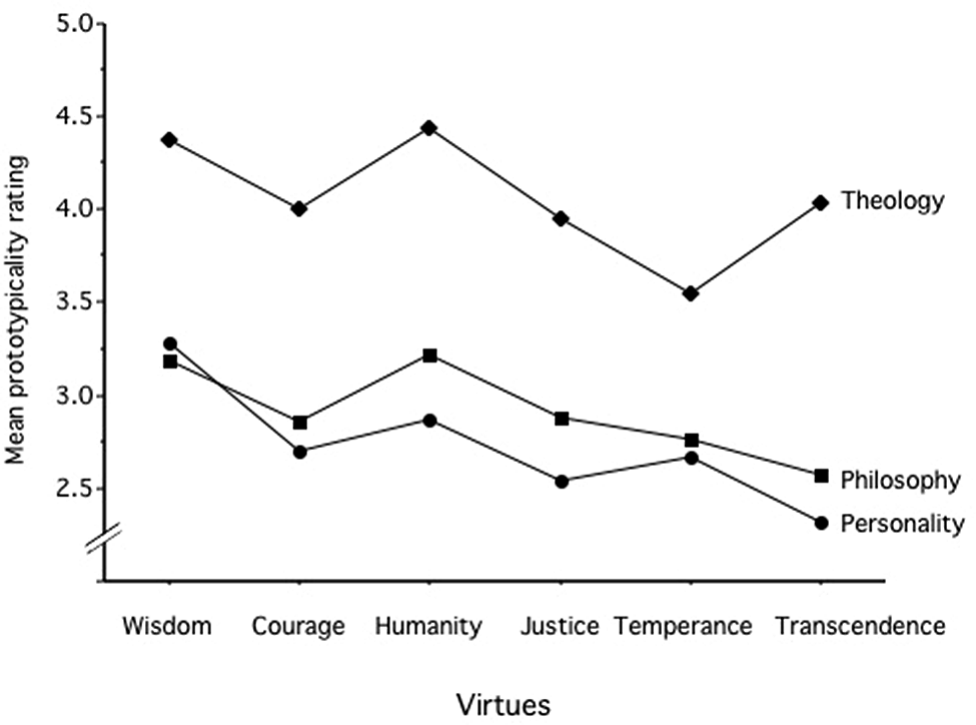
**Mean prototypicality rating for the 24 strengths in the six virtues for the groups of psychologists, philosophers, and theologians**.

A 3 × 6 × 24 ANCOVA with the expert type (psychologists, philosophers, theologians) as grouping factor, the six virtues and the 24 strengths on the repeated measure factors, and gender and age as covariates was computed for the prototypicality ratings. There was a strong effect of type of experts [*F*(1,61) = 20,681, *p* < 0.001, $\eta_{p}^{2}$ = 0.404], and the *post hoc* tests (Fishers PSL) showed that the theology group (*M* = 4.06) was higher (*p* < 0.001) than both the philosophers (*M* = 2.91) and psychologists (*M* = 2.73), which did not differ from each other (*p* = 0.55). Furthermore, type of expert was also involved in an interaction with virtue ($\eta_{p}^{2}$ = 0.102), strengths ($\eta_{p}^{2}$ = 0.057) and the virtue × strength interaction ($\eta_{p}^{2}$ = 0.093). Therefore, three separate 6 × 24 ANCOVAs were performed with the six virtues and the 24 strengths on the repeated measure factors, and gender and age as covariates were computed for the prototypicality ratings for the three groups. For the psychologists, the rating for wisdom was higher and the one for transcendence was lower than all others. Furthermore, humanity was rated higher than justice and temperance. For philosophers, both humanity and wisdom were higher than all others, transcendence was lower than justice, but not significantly lower than courage and temperance. For the theology group there were three clusters of virtues that differed from each other but not within: temperance was lower than all other virtues, humanity and wisdom were highest and courage, justice, and transcendence were in between (see **Figure 1**).

Furthermore, the interactions between virtues and strengths were all significant at *p* < 0.001 ($\eta_{p}^{2}$: psychologist = 0.132, philosophers = 0.129, and theologians = 0.075). Analyses like the one in **Table 5** were performed for the three groups separately (The table is available upon request). A count of proper and false assignments was undertaken. Overall, 96.7% of the strengths were assigned properly (see **Table 5**) and there were 24.0% of the cases where a strength exceeded 3.5 for a certain virtue. This analysis illuminated the rating behaviors of the three groups. The theologians did have a 100% hit rate for the assigned virtues, but also had 76.6% “false” assignments. Psychologists (75.00%) and philosophers (83.33%) did not judge all strengths being prototypical for the assigned virtue, but they also did not make many “false” assignments (psychologists: 9.27% and philosophers: 13.02%). In other words, the theology group erred on the overinclusion side: they did not miss any strength linked to a virtue, but saw many strengths linked to many virtues. Most strikingly, they saw each strength exceeding the prototypicality cut-off point for both wisdom and humanity. The psychologists and philosophers only did catch two out of the five transcendence strengths and also missed one (or two) strengths of justice. They did agree on what is a second marker. Wisdom also covered social intelligence and prudence, humanity covers teamwork, forgiveness, and gratitude, and courage also covered leadership.

### The Structure of Strengths as Reflected in the Virtue Prototypicality Ratings

Next, the 24 strengths were intercorrelated across raters and virtues (111 × 6 = 666 data points) simultaneously and subjected to a principal component analysis. Five Eigenvalues were greater one and the scree test suggested the retention of 5 or 6 factors (Eigenvalues: 8.25, 2.90, 1.93, 1.80, 1.41, 0.82, 0.68, 0.65, and 0.51). Both solutions were inspected and the six-factor solution (which explained 71.3% of the variance) was found to be more meaningful. The Varimax-rotated factors are given in **Table 6**.

**Table 6:** Varimax loadings of the 24 strengths of the six factors based on the analysis of the prototypicality ratings.

	Factor 1	Factor 2	Factor 3	Factor 4	Factor 5	Factor 6
Creativity	0.04	**0.73**	**0.41**	-0.03	-0.11	0.11
Curiosity	0.09	**0.78**	0.35	0.02	0.02	0.14
Judgment	0.04	**0.63**	0.18	0.19	**0.51**	0.01
Love of learning	0.11	**0.84**	0.14	0.18	0.04	0.08
Perspective	0.13	**0.66**	-0.10	0.26	**0.40**	0.20
Bravery	0.05	0.11	**0.86**	0.02	0.15	0.00
Endurance	0.03	0.26	**0.54**	**0.56**	0.02	-0.14
Honesty	0.24	0.16	**0.54**	0.11	**0.50**	0.08
Zest	0.10	0.29	**0.77**	0.01	0.01	0.22
Love	**0.85**	0.10	0.14	0.04	-0.07	0.07
Friendliness	**0.83**	0.03	0.13	-0.01	0.16	0.20
Social intelligence	**0.54**	0.37	0.04	0.17	**0.50**	0.00
Teamwork	**0.65**	0.03	0.08	0.19	0.36	-0.11
Fairness	**0.40**	-0.06	0.14	0.10	**0.75**	0.03
Leadership	0.26	0.24	**0.47**	0.10	**0.44**	-0.25
Forgiveness	**0.79**	0.07	0.02	0.16	0.18	0.13
Modesty	**0.43**	0.03	0.02	**0.67**	0.11	0.29
Prudence	0.09	0.30	-0.11	**0.71**	0.25	0.06
Self-regulation	0.09	0.01	0.13	**0.88**	0.02	0.03
Beauty/excellence	0.17	**0.47**	0.07	0.01	-0.01	**0.69**
Gratitude	**0.64**	0.11	0.04	0.15	0.32	**0.40**
Hope	0.22	0.22	**0.59**	0.05	0.01	**0.48**
Humor	**0.54**	**0.46**	0.24	0.07	0.06	0.17
Spirituality	0.18	0.07	0.11	0.12	-0.01	0.**82**

Loadings ≥ 0.40 are shown in boldface.

The factor of wisdom and knowledge explained 15.2% of the variance and not only encompasses creativity, curiosity, judgment, love of learning, and perspective, but also and to a lower extent appreciation of beauty and excellence, humor, and social intelligence. The latter three had double loadings and were also marking other factors. The factor of courage (12.5%) was clearly loaded by bravery and zest, and to a lower extent by endurance, and honesty, which also loaded on the factors of temperance and justice, respectively. Hope, leadership, and creativity also loaded on courage—all of them demonstrated double loadings. While the temperance strengths of modesty, prudence, and self-regulation were complemented by endurance, temperance (9.8%) was not loaded by forgiveness. Forgiveness (and partly also modesty), the strengths of humanity (love, kindness, and to a lower extent social intelligence), some strengths of justice (teamwork, fairness) marked the first and strongest (17.0% explained variance) factor together with gratitude and humor. Furthermore, a fifth factor resembles justice and was composed of fairness and leadership, but also judgment, perspective, honesty, and social intelligence. Finally, a factor of transcendence (8.1%) was clearly marked by spirituality and beauty/excellence and to a lower extent also by hope and gratitude. All except spirituality had double loadings. Humor was clearly not part of transcendence.

The labeling of the factors was underscored by the fact that the mean prototypicality ratings (see **Table 5**) and the factor loadings (**Table 6**) were very highly correlated. The more a strength was seen to represent a virtue, the higher was its loading on a factor labeled after this virtue. The coefficients were particularly high for humanity (*r* = 0.95), courage (*r* = 0.91), temperance (*r* = 0.90), and lower for justice and transcendence (both: *r* = 0.95), and somewhat lower for wisdom (*r* = 0.79; all *p* < 0.001). The coefficients between the non-homologous variables were between *r* = -0.50 and *r* = 0.36.

## Discussion

The present rating study provides support for the internal structure of the VIA-classification of virtues and strengths but also allows suggesting some changes. Most importantly, the scales are indeed prototypical for the virtues with 10 exceeding 5.0 (i.e., being very good markers), nine strengths exceeding 4.0 (i.e., being good markers, and four exceeding 3.5 (i.e., being a marker) of the virtue it was originally assigned to. Only one strength was not considered prototypical: humor did fail to reach the cut-off value of 3.5 for transcendence. Thus, the major outcome of the study is that the assignment of the strengths to virtues as put forward by Peterson and Seligman (2004) was correct with one exception. For humor one might consider relocating this strength under humanity, but it also shows relations to wisdom (Beermann and Ruch, 2009a,b).

The second major outcome is that several strengths relate to more than one virtue and occasionally they were found more prototypical for a different virtue then for the one they were assigned to. In fact, the major difference between high and low virtue strengths is the number of strengths they are prototypical for (not only the degree of prototypicality). This has at least two consequences. First, the fact that strengths may be prototypical for more than one virtue means that rotation to simple structure inevitably will not be successful, as simple structure expects variables to have a salient loading on only one factor, and zero loadings on the other factors. Second, this has implications for the theoretical model. Is a bi- or multimodal classification feasible? Is it compatible with Linnaean thinking? Can one say that the virtue of wisdom may not only be achieved through creativity, curiosity, or perspective but also through social intelligence and prudence? And the former also fosters humanity and the latter temperance? Shall the strengths that were more prototypical for a different virtue be rearranged in the classification and handbook (Peterson and Seligman, 2004); i.e., shall leadership be moved to courage, and teamwork, forgiveness, and gratitude be subsumed under humanity? There is some convergence across the prototypicality ratings and the results of the factor analysis and these converging deviations do provide a basis for starting to think about rearranging the entries of the classification. The present study only provides an initial step and more converging evidence needs to be accumulated.

So far, work on the structure of the strengths and virtues have been exclusively done in a factor-analytic framework. In such articles the authors typically insinuate that Peterson and Seligman (2004) had a hierarchical factor analytical model in mind just as it is omnipresent in personality research. Without giving any factual evidence (i.e., a direct quote from the book or articles) for this insinuation, authors typically proceed to say that they want to test this assumption (attributed to Peterson and Seligman) empirically. Virtually everyone failed to confirm what would be a factor analyst’s dream, namely, that the strengths intercorrelate in a way that there is a need to extract six factors, which can be identified as the postulated virtues and are loaded highly by the set of strengths assigned to that virtue (and only by those strengths). Based on both our theoretical reasoning and on the results of the present study we argue that running factor analyses on the instruments measuring the VIA classification is likely to face three challenges: first, studying the strengths-virtue relations by identifying the structure derived from the intercorrelation of the 24 strengths is bound to fail as the VIA-classification is not a factor-analytic model and virtues cannot be defined by the intercorrelation of strengths. Second imposing simple structure on the derived factor matrices is comparable to forcing the data into a Procrustean bed; i.e., fitting into an unnatural scheme or pattern. There is also a third problem that impairs finding a proper structure using factor analysis, namely, strengths of justice and strengths of humanity are more difficult to separate as the former are strengths *among*, and strengths of love are strengths *between* people, but the difference is assumed to be more of degree than of kind. Thus, it is unlikely to find the strengths loading on different factors. Perhaps the justice strengths jointly should fit under humanity; they should form one factor in a first step that then is—with the other strengths of humanity—a joint factor of humanity. Indeed, in the present study all strengths of justice were also covered by humanity but not vice versa, humanity strengths were not covered by justice; only social intelligence shows some fairly good prototypicality for justice. Here more theoretical work is needed that then can lead to empirical testing.

While we argue that factor analysis is not ideally suited to test the relation between strengths and virtues we do not imply that factor analyses of the strengths should not be conducted. It allows finding redundancy in the scales and it is also interesting to talk about the latent structure underlying the strengths—or the strengths as represented in measurement instruments. However, we do warn of two consequences: first, factors should not be expected to lead to the six virtues in the VIA classification. More importantly, we believe that it would be wrong to take such factors as the new reference and start deleting or adding strengths to fit these arbitrary factors.

The present study shows that humor might be the only clearly misclassified strength as there is no apparent link to transcendence. The pilot study showed that the five domains of descriptions converged very strongly for humor. Hence, this cannot be merely attributed to, for example, the items, which do not emphasize the transcendence aspect strongly enough. In fact, this was foreseen by Peterson and Seligman (2004) when stating: “In a few cases, the classification of a given strength under a core virtue can be debated. Playfulness, for example, might be considered a strength of humanity because humor and whimsy can create social bonds. It might also be classified as a wisdom strength, inasmuch as playfulness helps us acquire, perfect, and use knowledge. But we had a reason for dubbing playfulness a strength of transcendence: like hope and spirituality, playfulness connects us to something larger in the universe, specifically the irony of the human condition, the incongruent congruencies to which playful people call our attention, for our education and amusement” (p. 26). The results of the present study suggest that humor (as defined in the VIA-classification) is linked to both humanity and wisdom (to a lower extent). This underscores the results of prior studies that suggest that while humor may be related to all virtues, the alignment is most strongly for humanity and wisdom (Beermann and Ruch, 2009a,b; Müller and Ruch, 2011). This is the last issue to be highlighted in improving the fit between strengths and virtues. One should consider for each strength whether it can serve only one or more virtues, and if yes whether this should enter their definition. For example, humor may reflect wisdom when the incongruities people encounter in life are highlighted and this then enables a person not to take things too seriously. Benevolent humor serves humanity when we use it to brighten someone’s day or when making others laugh at our misfortunes. Humor in the form of understatement, especially as an alternative to being upset, and moderated statements that do not hurt anyone is in line with temperance. Satire and corrective humor may serve justice when we correct a bully or oppressor and protect a victim or target. Humor reflecting the insight that from a larger perspective our problems are minimal and as humans we are all bound to fail and we should be accepting this might relate to transcendence. So, at least for humor, the exact definition of the strength may be varied to match the fit to a virtue.

This study has several limitations. The selection of the experts and laypersons can be debated. We selected the experts based on their expertise, but cannot exclude that a different set of experts would have come to different conclusions. For example, the experts from theology where mostly chairs in catholic or protestant theology and maybe it would have been different if other religions had dominated. Also all experts were form the German speaking countries. Therefore, a cross-validation of the findings with a different set of raters form different parts of the world might be desirable. We had roughly the same amount of experts in personality, philosophy, and theology and hence the results are balanced across these disciplines. However, it became obvious that theologians were very different from psychologists and philosophers. They saw more of the strength related to virtues and did not discriminate that much among the virtues. This might be partly due their training and convictions but it might also be due to less familiarity with rating scales. Clearly, in particular the results for theologians await replication albeit the provided definitions of the virtues should minimize the subjectivity. We had the experts do one judgment and did not repeat it, so we do not know how stable these ratings are.

Another shortcoming might be the definition of the virtues. The core elements of the descriptions provided by Peterson and Seligman (2004) were used. Allowing for a more extensive study of the virtues might have been helpful, in particular to those that were not that familiar with virtues, or particular virtues, such as transcendence. Interestingly, also the laypersons did do comparably well and yielded results that were comparable to the experts, which means that the task was not that difficult. A replication study might allow for more time to get familiar with the virtues and maybe this will enhance the validity.

## Conclusions

The links between strengths and virtues as suggested by the present study are different from both the original classification (Peterson and Seligman, 2004) and the factor analytic studies (e.g., McGrath, 2014). The lesson to be learned is that the strengths may be in the service of several virtues simultaneously. This is not really in contradiction to the classification. If these double relations are a problem, then the concept needs to be purified and the contents that provide the link to the other virtues need to be stripped off. Furthermore, the results also demonstrate that studying the intercorrelation of the strengths is not the golden road to arrive at virtues. From the intercorrelation of strengths, a strength factor can be derived, but not necessarily a virtue. While individuals may act in accordance with a virtue to a certain extent (and thus produce individual differences in a virtue), the virtues are not defined by their mutual overlap among the strengths. The virtues exist independent of the different strengths that enable individuals to display that virtue. There is a body of research that defines virtue factors though intercorrelations of lexical virtue terms (e.g., De Raad and Van Oudenhoven, 2011). There are factors such as sociability, achievement, respectfulness, vigor, altruism, and prudence (De Raad and Van Oudenhoven, 2011) or self-confidence, reflection, serenity, rectitude, perseverance and effort, compassion, and sociability (Morales-Vives et al., 2014). Without going into detail, it can be seen that these virtues do not seem to be of a more narrow nature compared to “humanity” or “wisdom” and are more at the level of strengths. Likewise the shorter lists still are more narrow, representing empathy, order, resourcefulness, and serenity (Cawley et al., 2000). Therefore, it seems that the six core virtues of Dahlsgaard et al. (2005) are only partially directly represented in the virtue factors identified in the present study.

## Conflict of Interest Statement

The authors declare that the research was conducted in the absence of any commercial or financial relationships that could be construed as a potential conflict of interest.

## Footnotes

1 The use of this criterion over others (e.g., whether this strength is a route to displaying a virtue, or a means for achieving virtue) was recommended by Peterson (2006).
